# MR imaging in discriminating between benign and malignant paediatric ovarian masses: a systematic review

**DOI:** 10.1007/s00330-019-06420-4

**Published:** 2019-09-16

**Authors:** Lotte W. E. van Nimwegen, Annelies M. C. Mavinkurve-Groothuis, Ronald R. de Krijger, Caroline C. C. Hulsker, Angelique J. Goverde, József Zsiros, Annemieke S. Littooij

**Affiliations:** 1 Princess Máxima Center for Pediatric Oncology, Heidelberglaan 25, 3584 CS Utrecht, The Netherlands; 2 Department of Pathology, University Medical Center Utrecht, Utrecht, The Netherlands; 3 Department of Reproductive Medicine and Gynaecology, University Medical Center of Utrecht, Utrecht, The Netherlands; 4 Department of Radiology and Nuclear Medicine, University Medical Center Utrecht, Utrecht, The Netherlands

**Keywords:** Ovarian neoplasms, Magnetic resonance imaging, Systematic review

## Abstract

**Objectives:**

The use of magnetic resonance (MR) imaging in differentiation between benign and malignant adnexal masses in children and adolescents might be of great value in the diagnostic workup of sonographically indeterminate masses, since preserving fertility is of particular importance in this population. This systematic review evaluates the diagnostic value of MR imaging in children with an ovarian mass.

**Methods:**

The review was made according to the PRISMA Statement. PubMed and EMBASE were systematically searched for studies on the use of MR imaging in differential diagnosis of ovarian masses in both adult women and children from 2008 to 2018.

**Results:**

Sixteen paediatric and 18 adult studies were included. In the included studies, MR imaging has shown good diagnostic performance in differentiating between benign and malignant ovarian masses. MR imaging techniques including diffusion-weighted imaging (DWI) and dynamic contrast-enhanced (DCE) imaging seem to further improve the diagnostic performance.

**Conclusion:**

The addition of DWI with apparent diffusion coefficient (ADC) values measured in enhancing components of solid lesions and DCE imaging may further increase the good diagnostic performance of MR imaging in the pre-operative differentiation between benign and malignant ovarian masses by increasing specificity. Prospective age-specific studies are needed to confirm the high diagnostic performance of MR imaging in children and adolescents with a sonographically indeterminate ovarian mass.

**Key Points:**

*• MR imaging, based on several morphological features, is of good diagnostic performance in differentiating between benign and malignant ovarian masses. Sensitivity and specificity varied between 84.8 to 100% and 20.0 to 98.4%, respectively.*

*• MR imaging techniques like diffusion-weighted imaging (DWI) and dynamic contrast-enhanced (DCE) imaging seem to improve the diagnostic performance.*

*• Specific studies in children and adolescents with ovarian masses are required to confirm the suggested increased diagnostic performance of DWI and DCE in this population.*

**Electronic supplementary material:**

The online version of this article (10.1007/s00330-019-06420-4) contains supplementary material, which is available to authorized users.

## Introduction

Ovarian malignancies in children and adolescents are relatively rare, with an incidence of 3 per 100,000 compared with 56 cases per 100,000 at the age of 65 to 69 years [1–3]. Despite this low incidence, ovarian tumours constitute the most common gynaecological malignancy in children and adolescents. Paediatric ovarian masses encompass a variety of benign and malignant tumours, including rare types such as sex cord-stromal tumours [4–6]. Both this heterogeneity and the importance of fertility preservation in this age group make the diagnostic assessment of these masses challenging.

While malignant ovarian neoplasms may need a more aggressive surgical approach, benign masses can either be safely monitored or undergo simple resection allowing for a fertility- and ovary-sparing approach [7]. Being able to discriminate between benign and malignant masses of the ovary is therefore of considerable clinical importance in the initial surgical management [4, 8]. Ultrasound is the first imaging modality in the diagnostic assessment of ovarian masses at any age. Clinically useful rules have been established by the International Ovarian Tumor Analysis (IOTA) group to differentiate between benign and malignant masses. Nevertheless, in about one-fifth of the cases, the nature of the ovarian mass remains undefined [9].

**Fig. 1:**
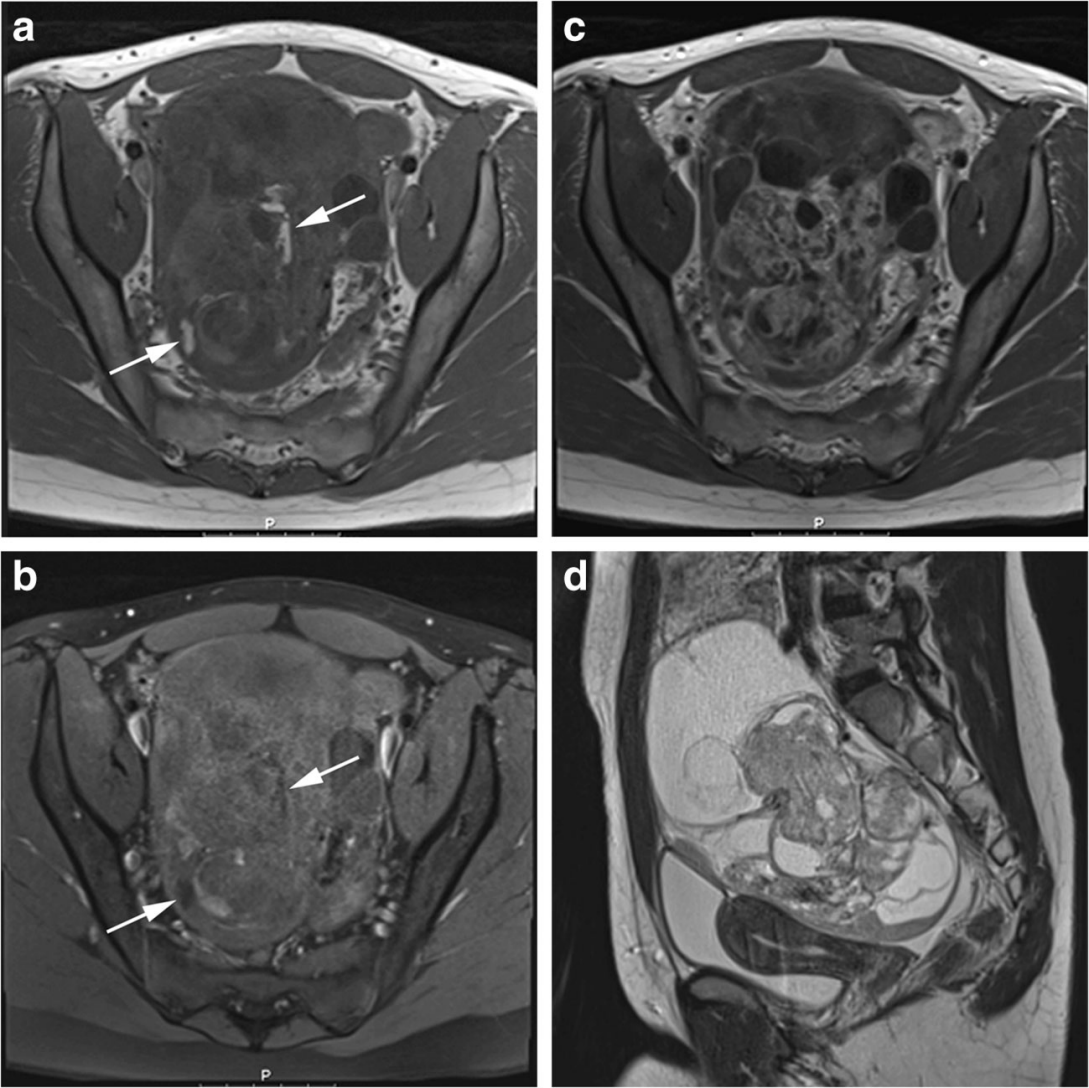
An example of immature teratoma grade 1 of the right ovary in a 15-year-old girl, treated as a benign tumour with local resection and follow-up. Axial T1-weighted before and after administration of gadolinium contrast (**a**, **c**), axial T1-weighted with fat-suppression (**b**) and sagittal T2-weighted turbo spin echo (**d**) show a cystic-solid mass with fatty components (arrows). Intralesional fat is diagnostic for a teratoma. The relative large amount of enhancing parts increases the risk of immature components

**Fig. 2:**
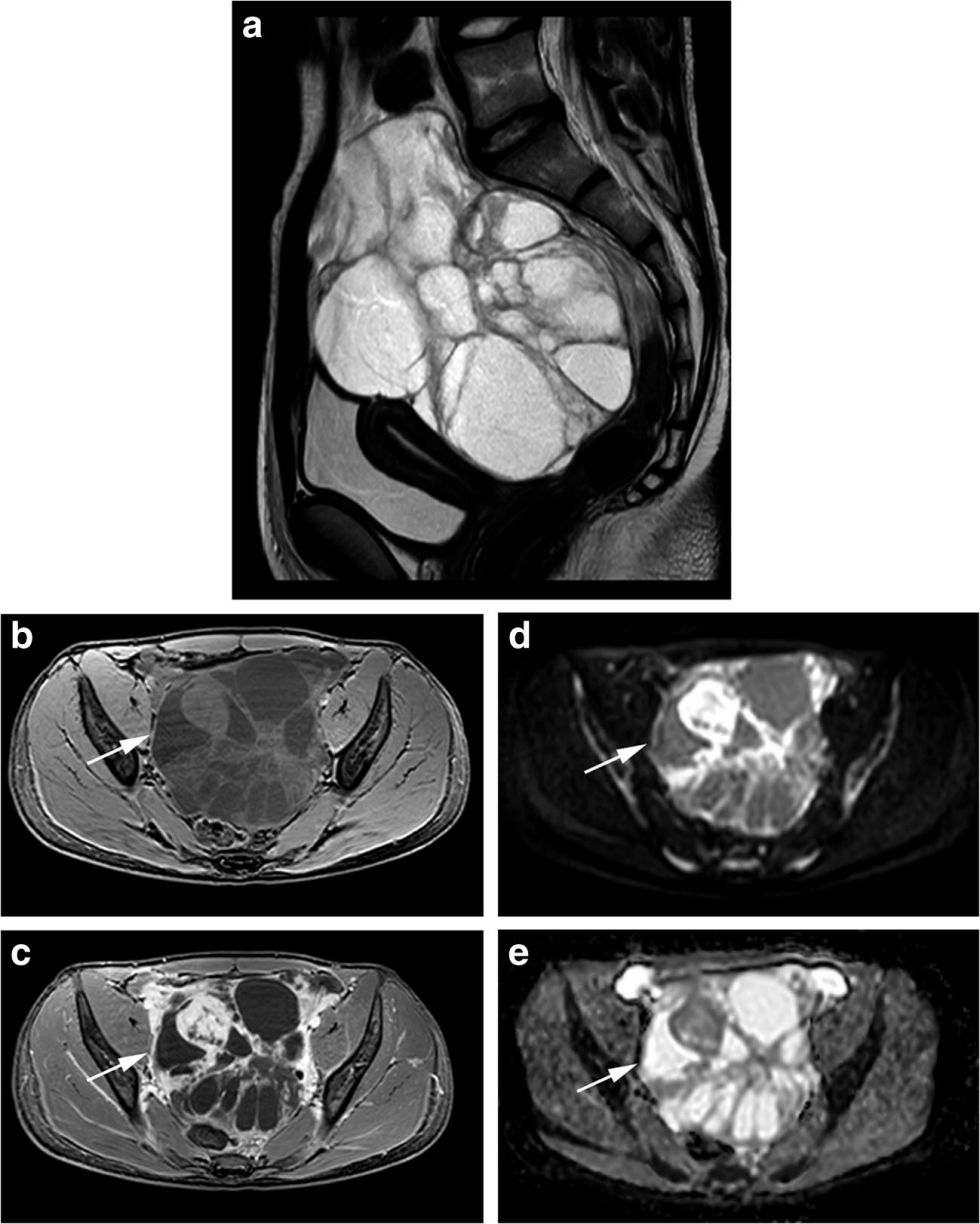
An example of yolk sac tumour of the right ovary in a 16-year-old girl. Sagittal T2-weighted turbo spin echo TSE (**a**) and T1-weighted gradient echo with fat suppression before and after administration of gadolinium contrast (**b**, **c**) show a large cystic solid mass in the lower abdomen. The enhancing parts of the lesion show relative impeded diffusion (arrow) at axial DWI (b1000 and ADC map; **d**,**e**)

In case of sonographically indeterminate ovarian masses, magnetic resonance (MR) imaging can provide additional information, e.g. on the different components of the mass, tumour rupture and peritoneal depositions. Figures 1 and 2 show examples of an immature teratoma grade I (treated as a benign tumour with local resection and follow-up) and a malignant yolk sac tumour. Functional imaging techniques like diffusion-weighted imaging (DWI) and dynamic contrast-enhanced (DCE) imaging could be of additional value [10]. DCE enables qualitative, quantitative or semi-quantitative evaluation of tumour vascularity, thereby providing information about the nature of the mass. This investigation is based on enhancement patterns, expressed as time-intensity curves (TICs), of which three different types are acknowledged. Type I displays a gradual, continuous rise in signal intensity; type II shows a moderate rise in signal intensity followed by a plateau; and type III is characterised as early washout [11, 12]. In adults, several studies have evaluated the diagnostic value of MR imaging in differentiating between malignant and benign neoplasms and characterising the specific nature of ovarian masses. Based on these studies, the European Society of Urogenital Radiology (ESUR) has developed an algorithmic approach for the imaging of the sonographically indeterminate adnexal mass [7, 13–16]. However, data on the role of MR imaging in discriminating between benign and malignant ovarian masses in children is scarce. In this systematic review, we evaluate the diagnostic value of MR imaging in children and adolescents with an ovarian mass, including the value of additional MR techniques.

## Methods

### Search strategy and eligibility criteria

This review is written according to the PRISMA Statement [17]. A thorough search of PubMed and EMBASE for all available literature published from 2008 to 2018 was performed. These libraries were systematically searched for original studies on the use of MR imaging in differential diagnosis of ovarian masses in both adult women and children. We classified studies into two groups. Studies were classified as ‘paediatric’, when the age of all included patients was 18 years or less. Studies performed on adult women, on the other hand, were classified as ‘adult’. The full search strategy is provided in Supplementary Table 1. Articles were included if suspected ovarian masses were evaluated with MR imaging (either 1.5 T or 3.0 T), including the evaluation of contrast enhancement, and were compared with a histopathology reference standard. Studies providing no description of MR imaging findings and studies on adult women that analysed selectively benign, borderline or malignant masses were excluded. However, similar studies as well as case reports performed on paediatric patients were included, in order to minimise the risk of missing relevant studies. Since ovarian carcinomas are very rare in children, only studies performed on adult patients that included more than 20% of malignant tumours other than carcinoma were considered relevant for this review. This particular cut-off was chosen pragmatically, since it was expected most MR studies in adult ovarian tumours focus on epithelial neoplasms, due to its prevalence of 80–90%.

All studies resulting from the literature search were assessed independently by two researchers (A.M., L.N.). Disagreements about study inclusion or exclusion were settled by consensus.

### Quality assessment

The quality of the individual studies was judged using the “Standards for Reporting Diagnostic Accuracy 2015” (STARD 2015) checklist [18]. Included studies were further assessed for methodologic quality independently by two researchers (A.M., L.N.), using the Oxford Centre for Evidence-Based Medicine Levels of Evidence Classification rubric [19].

### Data extraction

From the included studies, population size expressed as the number of ovarian masses analysed, mean age of the participating patients, histopathological classification of the ovarian masses and MR imaging protocol and analysis, as well as MR imaging features of the concerning ovarian masses, were scored. As for MR imaging features, information about the following parameters were extracted: size, shape, boundary, wall and septum thickness, vegetation, mass configuration, bilaterality, signal intensity of T1-weighted imaging, ascites/pelvic fluid, peritoneal implants/nodules and contrast enhancement. If available, information on *b*-values used in DWI and apparent diffusion coefficient (ADC) values were collected. Concerning semi-quantitative DCE, data on TICs, enhancement amplitude and time to peak were included. Lastly, data on diagnostic performance expressed as sensitivity, specificity, accuracy, positive predictive value (PPV), negative predictive value (NPV) or area under the curve (AUC) for these individual parameters were extracted when provided.

## Results

### Search strategy and eligibility criteria

**Fig. 3:**
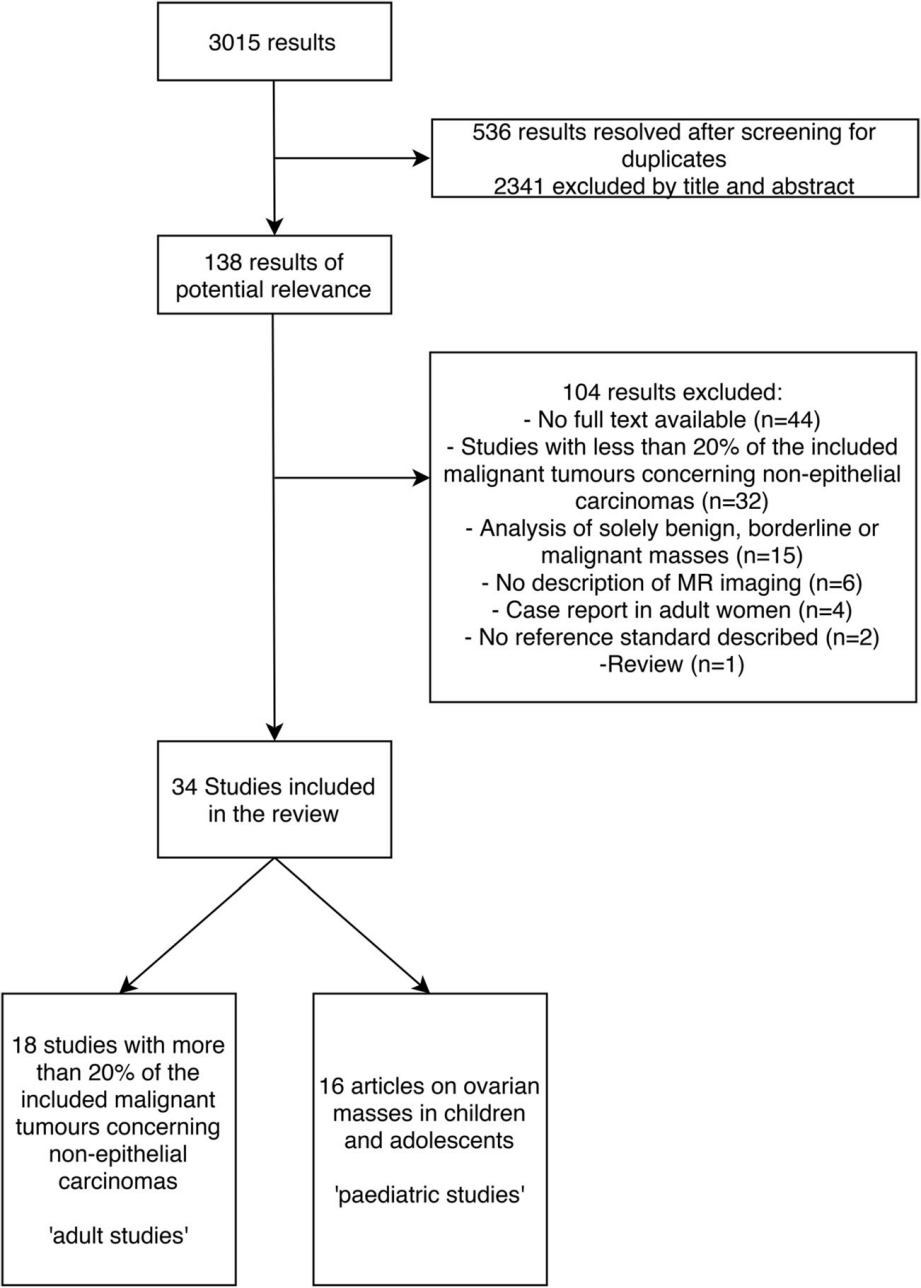
The flowchart summarises the search process with the number of studies included and excluded

The study selection process is shown in Fig. 3. The search in PubMed and EMBASE resulted in 3015 studies, of which 536 studies turned out to be duplicates. The remaining 2479 studies were screened by title and abstract, based on which 2341 studies were excluded. Consequently, 138 articles were of potential relevance to this systematic review and their full texts were analysed. This led to the exclusion of another 104 studies. The remaining 34 studies were analysed in this review.

### Quality assessment

The studies in adult women were predominantly scored as Oxford Evidence level 2 (cross-sectional studies with consistently applied reference standard and blinding). Levels of evidence of the individual studies can be found in Table 1. Quality assessment of the included studies in adult women, using the STARD 2015, is provided in Supplementary Table 2.

**Table 1:** Characteristics of the studies included in this systematic review regarding the use of MR imaging in differential diagnosis of ovarian masses

Study (reference)	Oxford level	*n*	Mean age in years	Histopathological classification of included ovarian masses
Adult studies				
Li et al 2017 [11]	2	102	57 (benign), 37 (borderline), 54 (malignant)	Benign (*n* = 15), borderline (*n* = 16), malignant (*n* = 71)
Li et al 2015 [12]	3	48	NA (range 11–79)	Benign (*n* = 13), malignant (*n* = 35)
Zhao et al 2018 [20]	3	42	52 (benign), 41 (malignant)	Benign (*n* = 29), malignant (*n* = 13)
Zhang et al 2012 [21]	2	139	52	Cysts (*n* = 21), endometriomas (*n* = 33), benign (*n* = 43), malignant (*n* = 42)
Bernardin et al 2012 [22]	2	67	48	Benign (*n* = 31), malignant (*n* = 36)
Nasr et al 2014 [23] 3		23	36 (benign), 45 (malignant)	Benign (*n* = 12), malignant (*n* = 11)
Takeuchi et al 2009 [24]	2	49	59	Benign (*n* = 10), borderline (*n* = 6), malignant (*n* = 33)
Mansour et al 2015 [25]	2	235	39	Benign (*n* = 75), malignant (*n* = 160)
Zhang et al 2012 [26]	3	202	57	Benign (*n* = 74), malignant (*n* = 128)
Tsili et al 2008 [27]	2	89	67	Benign (*n* = 66), malignant (*n* = 23)
Dilks et al 2010 [28]	2	26	43	Benign (*n* = 14), malignant (*n* = 12)
Tsuboyama et al 2014 [29]	2	127	53	Benign (*n* = 30), borderline (*n* = 31), malignant (*n* = 66)
Elzayat et al 2017 [30]	3	32	39 (benign), 34 (borderline), 43 (malignant)	Benign (*n* = 7), borderline (*n* = 4), malignant (*n* = 21)
Emad-Eldin et al 2018 [31]	2	65	44	Benign (*n* = 30), borderline (*n* = 7), malignant (*n* = 28)
Mansour et al 2015 [32]	2	150	29 (benign), 39 (borderline), 46 (malignant)	Benign (*n* = 42), borderline (*n* = 26), malignant (*n* = 82)
Li et al 2018 [33]	2	109	57 (benign), 34 (borderline), 51 (malignant)	Benign (*n* = 15), borderline (*n* = 28), malignant (*n* = 66)
Zhang et al 2014 [34]	2	144	37 years (endometric cysts), 40 years (teratomas)	Endometric cysts (*n* = 35), teratomas (*n* = 28)
Zhao et al 2014 [35]	2	50	51 (benign), 41 (borderline)	Benign (*n* = 26), borderline (*n* = 24)
Paediatric studies				
Emil et al 2017 [36]	3	18	15	Benign
Marro et al 2016 [37]	2	32	13	Benign, borderline and malignant
Thomas et al 2012 [38]	4	1	14	Bilateral mucinous cystadenomas
Willems et al 2012 [39]	4	1	15	Benign mucinous cystadenoma
Park et al 2010 [40]	4	1	11	Sclerosing stromal tumour
Ghanbari 2013 [41]	4	1	3	Juvenile granulosa cell tumour
Tsuboyama et al 2018 [42]	4	2	14 (1), 10 (2)	Dysgerminoma
Bedir et al 2014 [43]	4	1	10	Juvenile granulosa cell tumour
Boraschi et al 2008 [44]	4	1	7	Immature teratoma
Chaurasia et al 2014 [45]	4	1	7	Sclerosing stromal tumour
Lin et al 2017 [46]	4	74	6	Germ cell tumours
Pollmann et al 2017 [47]	4	1	13	Mature teratoma
Braun et al 2012 [48]	4	1	12	Leydig cell tumour
Calcaterra et al 2013 [49]	4	1	8	Juvenile granulosa cell tumour
Rogers et al 2014 [50]	4	129	12	Benign and malignant
Nejkovic et al 2012 [51]	4	1	17	Mature teratoma

Characteristics of all studies included in this systematic review, including the number of ovarian masses analysed, histopathological classification hereof and mean age of the participants per concerning study. The methodologic quality of included studies based on the Oxford Centre for Evidence-Based Medicine Levels of Evidence Classification rubric is provided as well

*n =* population size expressed as number of ovarian masses included in the original study

*NA*, not available

Since most studies in children and adolescents concerned either case reports or case series, the majority of these were scored as Oxford Evidence level 4, with the exception of two studies (one cross-sectional study, one non-consecutive study) (Table 1).

### Characteristics of included studies

The characteristics of the included studies (18 ‘adult’ [11, 12, 20–35] and 16 ‘paediatric’ studies [36–51]) are provided in Table 1. The mean age of patients included was 10.8 years in the paediatric and 46.9 years in the adult studies. The number of ovarian lesions analysed ranged between 1 and 74 in the paediatric studies and between 23 and 235 in the adult studies. All studies analysed the use of MR imaging in differentiating between benign and malignant tumours of the ovary, with several studies incorporating the differentiation of epithelial borderline tumours as well.

### Paediatric studies

Table 2 shows MR imaging findings of the sixteen studies that were included: three cohort studies and 13 case reports. All three cohort studies analysed the diagnostic performance of MR imaging in children and adolescents with ovarian masses (or ovarian germ cell tumours specifically). The thirteen case reports describe limited data on MR characteristics.

**Table 2:** MR imaging descriptions of ovarian masses provided by ‘paediatric studies’

	Histopathological classification	Conventional MR imaging findings			DWI	DCE	Diagnostic performance							
Original studies														
Emil et al [36]	Benign	No report			No report	No report	Sensitivity, specificity, NPV, PPV and accuracy of MRI in characterising adnexal lesions as neoplastic: 89%, 94%, 94%, 89% and 93%, respectively							
Marro et al [37]	Benign, borderline and malignant	No report			No report	No report	‘MRI correctly suggested benign nature in 24/28 (85.7%) benign masses and was indeterminate for the nature in remaining 4 masses. MRI correctly suggested malignant nature in 3/4 malignant masses and was indeterminate in the mature teratoma with a microscopic focus of yolk sac tumour’							
Lin et al [46]	Germ cell tumours	No report			No report	No report	Sensitivity of MRI of 97%							
	Histopathological classification	Conventional MR imaging findings											DWI	DCE
		Size	Walls/septa	Vegetation	Boundary	Shape	Mass configuration	Bilaterality	T2WI	Ascites	Peritoneal implants	Constrast enhancement		
Case reports														
Thomas et al [38]	Bilateral mucinous cystadenomas	7 × 3 × 4 cm 9 × 5 × 5 cm 16 × 8 × 18 cm	No report	No report	Capsulated	No report	No report	No report	No report	No report	Absent	No report	No report	No report
Willems et al [39]	Benign mucinous cystadenoma	17.5 cm	No report	No report	Well-defined	No report	Multicystic	No report	No report	No report	Absent	Varying enhancements on FS T1WI	No report	No report
Park et al [40]	Sclerosing stromal tumour	8.9 × 2.6 × 6.6 cm	No report	No report	Well-defined	No report	No report	No report	No report	No report	No report	No report	No report	No report
Ghanbari [41]	Juvenile granulosa cell tumour	No report	No report	No report	No report	No report	Multiple cystic components	No report	No report	No report	Tumour adhesion to anterior bowel loops	No report	No report	No report
Tsuboyamaet al [42]	Dysgerminoma combined with gonadoblastomas	17 cm	Fibrovascular septa	Nodules (1.5 cm)	No report	Lobulated	No report	Absent	Intermediate signal intensity	No report	No report	Strong enhancement of fibrovascular septa on FS CE T1WI	High signal intensity	No report
	Yolk sac tumour and dysgerminomas with gonadoblastoma	± 8 cm	No report	Nodules (5 mm)	Smooth outlined	No report	No report	Absent	A homogeneous hyperintense area A heterogeneous mass intermediate to high signal intensity Nodules with intermediate intensity	No report	No report	Strong enhancement of the heterogeneous area and nodules on FS CE T1WI	High signal intensity of the heterogeneous mass and nodules	No report
Bedir et al [43]	Juvenile granulosa cell tumour	76 × 87 × 75 mm	No report	No report	No report	No report	Multiple cystic and solid components	No report	No report	No report	No report	No report	No report	No report
Boraschi et al [44]	Immature teratoma	6 × 7 × 7 cm	No report	No report	Capsulated	Round	Liquid (prevalent) and solid components (peripherally, ‘fat and a small signal void’)	Multicystic appearance of the other ovary	Iso- to hyperintensity of the solid component	Present	Absent	No report	No report	No report
Chaurasia et al [45]	Sclerosing stromal tumour	10 × 9 × 5 cm	No report	No report	Well-defined	No report	Heterogeneous solid and cystic mass	Absent	No report	No report	No report	No report	No report	No report
Pollmann et al [47]	Mature teratoma	28 × 19 × 12 cm	No report	Solid vegetation with calcifications	No report	No report	Cystic	Other ovary undetectable	No report	No report	No report	No report	No report	No report
Braun et al [48]	Leydig cell tumour	8 × 13 × 12 mm	No report	No report	No report	No report	No report	No report	No report	No report	No report	‘Absorbing the contrast agent’	No report	No report
Calcaterra et al [49]	Juvenile granulosa cell tumour	13 × 13 × 7.6 cm	No report	No report	No report	No report	No report	No report	No report	No report	No report	No report	No report	No report
Rogers et al [50]	Benign and malignant	No report	No report	No report	No report	No report	No report	No report	No report	No report	No report	No report	No report	No report
Nejkovic et al [51]	Mature teratoma	100 mm in diameter	No report	No report	No report	No report	Heterogeneous and cystic	Tumoural aspect of the other ovary	No report	No report	Absent	No report	No report	No report

Summary of the findings on MR, DW and DCE imaging by both original studies and case reports on ovarian masses in children and adolescents. Histopathological classification of the concerning masses and diagnostic performance of MR imaging, if available, are provided as well

*DWI*, diffusion-weighted imaging; *DCE*, dynamic contrast-enhanced imaging; *NPV*, negative predictive value; *PPV*, positive predictive value; *FS T1WI*, fat suppression T1-weighted imaging; *FS CE T1WI*, fat suppression contrast-enhanced T1-weighted imaging

### Adult studies

#### MR imaging

Ten studies provided a description of MR imaging features. The most often-described features (> 4 out of 10 studies) concerned size, thickness of walls and septa (when present), presence of vegetation, mass configuration, bilaterality, signal intensity on T2-weighted imaging, presence of ascites or peritoneal implants and contrast enhancement. An increased risk of malignancy was related to increased size of the lesion, increased wall thickness, presence and increased size of vegetation, mixed cystic and solid configuration, intermediate to high intensity on T2-weighted imaging, presence of contrast enhancement and of ascites or peritoneal implants. Six of the studies performed an analysis of the diagnostic performance of MR imaging [23, 27, 29–31, 34]. Criteria predictive of malignancy, sensitivity, specificity, PPV, NPV and accuracy, if provided, are depicted in Table 3. Sensitivity and specificity, depending on the criteria used, varied between 84.8 to 100% and 20.0 to 98.4%, respectively.

**Table 3:** Diagnostic performance of MR imaging in differential diagnosis of ovarian masses

Study (reference)	Criteria for malignancy	Diagnostic performance of MR imaging in differentiation between	Sensitivity	Specificity	PPV	NPV	Accuracy	AUC
Nasr et al [23]	Wall thickness > 3 mm Solid vegetation > 1 cm Thick septa > 3 mm Areas of necrosis and breaking down	Malignant and non-malignant	90.9%	58.3%	66.7%	87.5%	73.9%	NA
Tsili et al [27]	Primary features: • Presence of masses bilaterally • Size > 4 cm • Areas of necrosis • Partly cystic-solid mass configuration, with contrast enhancement of the solid components • Cystic or solid-cystic lesions with thick and irregular walls or septa, of thickness > 3 mm and/or papillary projections, demonstrating contrast enhancement Secondary features: • Pelvic organ or wall invasion, ascites, peritoneal metastases or lymphadenopathy • Characterised as malignant when two primary or one primary and one secondary feature present	Malignant and non-malignant	95.2%	98.4%	NA	NA	97.6%	NA
Tsuboyama et al [29]	Unilateral or bilateral masses with papillary projections or irregular solid portions showing intermediate intensity on T2WI	Benign and borderline/malignant	96.9% (93.5–100)	20.0% (5.7–34.3)	NA	NA	78.7% (71.6–85.9)	NA
		Benign/borderline and malignant	84.8% (76.2–93.5)	36.1% (24.0–48.1)			61.4% (53.0–69.9)	
Elzayat et al [30]	Wall thickness > 3 mm Solid vegetation > 1 cm Thick septa > 3 mm Areas of necrosis and breaking down	Malignant and non-malignant	92%	57.1%	88.4%	66.6%	84.4%	NA
Emad-Eldin et al [31]	Wall thickness > 3 mm Solid vegetation > 1 cm Thick septa > 3 mm Areas of necrosis and breaking down	Malignant and non-malignant	94.3%	90%	91.6%	93.1%	92.3%	NA
Zhang et al [34]	Vegetation and irregular thickened septa or walls of > 3 mm and solid components. In addition, any features of peritoneal or omental disease, lymphomas and ascites were considered criteria for malignancy	Malignant and non-malignant	92.7% (79.0–98.1)	89.3% (81.3–94.3)	77.6% (63.0–87.8)	96.8% (90.4–99.2)	90.3% (83.9–94.4)	97.2% (94.7–99.7)

Criteria used to assess the malignancy of ovarian masses on MR imaging and the diagnostic performance hereof, expressed as sensitivity, specificity, PPV, NPV, accuracy and AUC, if available

*PPV*, positive predictive value; *NPV*, negative predictive value; *AUC*, area under the curve; *NA*, not available

#### DWI-MR imaging

Eight studies investigated the value of DWI-MRI in the differential diagnosis of ovarian masses [20, 21, 23–26, 31, 34]. *b*-values (s/mm^2^), regions of interest (ROI) used to calculate ADC values (× 10^−3^ mm^2^/s) and diagnostic performance are shown in Table 4. Mean ADC values for benign and malignant lesions exhibited a significant overlap, with values for benign masses varying between 1.16 and 2.03 × 10^−3^ mm^2^/s, whereas the range of ADC values reported for malignant masses was 0.76 to 1.39 × 10^−3^ mm^2^/s. Three of the included studies provided information on the diagnostic performance of DWI [23, 25, 31]. Nasr et al provided diagnostic performance of DWI solely, with sensitivity, specificity and accuracy of 100%, 75% and 87% respectively [23]. Emad-Eldin et al and Mansour et al demonstrated sensitivity, specificity and accuracy of DWI additional to MR imaging of 100% and 93.3%; 96.77% and 85%; and 98.46% and 82.3% respectively [25, 31]. The diagnostic performance of specific ADC cut-off values, if provided, is shown in Table 4.

**Table 4:** Diagnostic performance of DWI-MR imaging in differential diagnosis of ovarian masses

Study (reference)	*b*-values (s/mm^2^) used	Region of interest (ROI)	ADC values (× 10^–3^ mm^2^/s)			Diagnostic performance						
			Benign	Borderline	Malignant	Measure	Sensitivity	Specificity	PPV	NPV	Accuracy	AUC
Zhao et al [20]	0 and 1000	Solid components	Benign SCSTs 1.343 ± 0.528*	NA	Malignant SCSTs 0.825 ± 0.129*	ADC 0.838	61.5%	89.5%	NA	NA	78.1%	NA
Zhang et al [21]	0 and 700	Both cystic and solid components	Benign 2.03 ± 0.94* (1)	NA	Malignant 1.39 ± 0.62* (1)	NA	NA	NA	NA	NA	NA	NA
Nasr et al [23]	0, 300 and 600	Both cystic and solid components	1.864 ± 0.585 (1)	NA	0.843 ± 0.165 (1)	DWI-MRI	100%	75%	79%	100%	87%	NA
Takeuchi et al [24]	0 and 800	Solid components	1.38 ± 0.30* (1)	NA	1.03 ± 0.19* (1)	ADC 1.15 ADC 1.0	74% 46%	80% 100%	94% 100%	44% 32%	NA	NA
Mansour et al [25]	0, 500, 1000 and 1500	Solid components	1.2 ± 0.34* (1)	1.1 ± 0.06^ (1)	0.83 ± 0.15*^ (1)	Conventional MRI + DWI	93.3%	85%	88.5%	94.4%	82.3%	NA
Zhang et al [26]	0 and 1000	Solid components	1.22 ± 0.46* (1)	NA	0.91 ± 0.20* (1)	ADC 1.20 ADC 1.20 (when cystadenofibromas, fibrothecomas and Brenner tumours are excluded)	66.7% 97.7%	90.9% 90.1%	81.4% 86.6%	82.1% 99.1%	NA	0.72 0.96
Emad-Eldin et al [31]	0, 500, 1000 and 1500	Solid components	1.16 ± 0.44 (1)	0.92 ± 0.38 (1)	0.76 ± 0.23 (1)	Conventional MRI + DWI ADC 0.95	100% 90.5%	96.77% 63.4%	97.14% 54.3%	100% 93.3%	98.46% 72.3%	NA
Zhang et al [34]	0 and 700	Unclear	2.0 ± 0.99 (1)	NA	1.36 ± 0.63 (1)	NA	NA	NA	NA	NA	NA	NA

Summary of the DWI protocols, including used *b*-values and regions of interest, and corresponding diagnostic performance of DWI imaging in ovarian masses. ADC values of benign, borderline and malignant masses, if available, are provided as well

*DWI*, diffusion-weighted imaging; *ADC*, apparent diffusion coefficient; *SCST*, sex cord-stromal tumour; *PPV*, positive predictive value; *NPV*, negative predictive value; *AUC*, area under the curve; *NA*, not available

*Statistically significant difference between benign and malignant with *p* value < 0.05

^Statistically significant difference between borderline and malignant with *p* value < 0.05

(1)Mean ADC value

#### DCE-MR imaging

Nine studies investigated the value of DCE-MRI in the differential diagnosis of ovarian masses [11, 12, 22, 23, 25, 28, 30–32]. Data on the TICs and semi-quantitative DCE parameters are depicted in Table 5, as well as diagnostic performance of this sequence and accompanying TICs. Five of these studies divided the different ovarian masses analysed by type of TIC. Type I TICs were most frequently found in benign lesions, with 33 to 85.7% of benign masses showing type I TICs. In type III TICs, on the other hand, there appeared more characteristics of malignancy, with 57.1 to 94.3% of all malignant masses exhibiting type III TICs. Overlap between benign and malignant masses was found by Elzayat et al [30] and Mansour et al [32], with one and nine malignant masses exhibiting a type I TIC, respectively. Overlap was also demonstrated by Li et al [12], with 3 benign masses exhibiting a type III TIC. The enhancement amplitude constituted one of the semi-quantitative parameters and was expressed in various ways, including maximum relative enhancement percentage (MRE%), maximum absolute enhancement (SImax), maximum relative enhancement (SIrel) and signal intensity at 60 s after enhancement (SI_60_). Malignant masses generally showed an increased enhancement amplitude compared with benign or borderline masses, with some of the studies demonstrating a statistically significant difference between these groups. Time to peak constituted the other semi-quantitative parameter and was indicated by time of half rising (THR), Tmax and time to peak within 200 s after enhancement (TTP_200_). All studies analysing this parameter agreed on malignant masses exhibiting a shorter time to peak compared with benign masses, again in several of these studies with statistically significant difference. Four studies provided information on the diagnostic performance of DCE [23, 25, 30, 31]. Nasr et al [23] and Elzayat et al [30] provided diagnostic performance of solely DCE, with sensitivity, specificity and accuracy of 60% and 80%; 91% and 100%; and 77.2% and 96%, respectively. Mansour et al [25] and Emad-Eldin et al [31] demonstrated sensitivity, specificity and accuracy of DCE in addition to MR imaging of 93.3 and 94.3; 100 and 100%; and 95% and 96.9%, respectively.

**Table 5:** Diagnostic performance of DCE-MR imaging in differential diagnosis of ovarian masses

Study (reference)	Time-signal intensity curves			Semi-quantitative parameters						Diagnostic performance						
	Benign	Borderline	Malignant	Enhancement amplitude			Time to peak			Measure	Sensitivity	Specificity	PPV	NPV	Accuracy	AUC
				Benign	Borderline	Malignant	Benign	Borderline	Malignant							
Li et al [11]	Type I: 5 (33%)* Type II: 10 (67%)* Type III: 0 (0%)*	Type I: 3 (19%)^ Type II: 9 (56%)^ Type III: 4 25%)^	Type I: 0 (0%)*^ Type II: 12 (17%)*^ Type III: 59 (83%)*^	EA 220.2 ± 90.5	EA 269.3 ± 70.9	EA 267.4 ± 86.2	THR 55.5 ± 15.4*	THR 37.3 ± 15^	THR 32.4 ± 8.5*^	TIC type III as indication for malignancy	83%	100%	NA	NA	86%	NA
Li et al [12]	Type I: 8 (61.5%)* Type II: 2 (15.4%)* Type III: 3 (23.1%)*	NA	Type I: 0 (0%)* Type II: 2 (5.7%)* Type III: 33 (94.3%)*	SI60 76.42 ± 32.82*	NA	SI60 129.17 ± 19.37*	NA	NA	TTP200 72.89 ± 22.69*	NA	NA	NA	NA	NA	NA	NA
Bernardin et al [22]	NA	NA	NA	Simax 491.2 ± 467.2* Sirel 55.4 ± 38.6*	Simax 360.2 ± 186.2^ Sirel 38.8 ± 22.1^	Simax 712 ± 278.6*^ Sirel 81 ± 33.5*^	NA	NA	NA	NA	NA	NA	NA	NA	NA	NA
Nasr et al [23]	NA	NA	NA	MRE% 73 ± 22.9	NA	MRE% 130 ± 27	Time to peak 92 ± 14.3	NA	Time to peak 53 ± 14.3	DCE-MRI	60%	91%	85%	73.2%	77.2%	NA
Mansour et al [25]	NA	NA	NA	NA	NA	NA	NA	NA	NA	Conventional MRI + DCE-MRI	93.3%	100%	100%	92.3%	95%	NA
Dilks et al [28]	NA	NA	NA	Simax 121 ± 184* Sirel 12.8 ± 19.2*	NA	Simax 589 ± 249* Sirel 101 ± 166*	NA	NA	NA	NA	NA	NA	NA	NA	NA	NA
Elzayat et al [30]	Type I: 4 (57%) Type II: 3 (43%) Type III: 0 (0%)	Type I: 2 (50%) Type II: 2 (50%) Type III: 0 (0%)	Type I: 1 (4.8%) Type II: 8 (38.1%) Type III: 12 (57.1%)	MRE% 89*	MRE% 115^	MRE% 168*^	Tmax 231*	Tmax 175	Tmax 119*	DCE-MRI in discrimination of benign versus borderline + malignant	80%	100%	100%	58%	96%	NA
Emad-Eldin et al [31]	Type I: 22 (73.3%)	Type I: 3 (42.9%)	Type I: 0 (0%) Type II: 7 (25%) Type III: 21 (75%)	Simax 704 ± 379.35 MRE% 76.15 ± 51.36	Simax 654 ± 356.3 MRE% 81.9 ± 52.29	Simax 1267 ± 503.5 MRE% 136.32 ± 54.8	Tmax 232 ± 92.58	Tmax 184.9 ± 53.04	Tmax 119 ± 43.97	Conventional MRI + DCE-MRI	94.3%	100%	100%	93.75%	96.9%	NA
Mansour et al [32]	Type I: 36 (85.7%) Type II: 6 (14.3%) Type III: 0 (0%)	Type I: 8 (30.8%) Type II: 8 (30.8%) Type III: 10 (38.4%)	Type I: 9 (11.0%) Type II: 22 (26.8%) Type III: 51 (62.2%)	MRE% 98.5 (65–158)*	MRE% 100 (81–124)^	MRE% 150.5 (144.5–222.5)*^	Tmax 278 (218.5–346)*#	Tmax 222 (183.5–302)#^	Tmax 138.5 (78–178.5)*^	SER type III as indication for malignancy: DCE-MRI in discrimination of benign masses DCE-MRI in discrimination of borderline masses DCE-MRI in discrimination of malignant masses	84.2%	85.7%	NA	NA	84.7% 76.2% 96.3% 77%	NA

Summary of the findings on and diagnostic performance of DCE imaging in ovarian masses. Both qualitative assessments by describing the time-signal intensity curves (TICs) and semi-quantitative assessments (various parameters) are demonstrated

*DCE*, dynamic contrast-enhanced imaging; *TIC*, time-signal intensity curve; *EA*, enhancement amplitude; *SI60*, signal intensity at 60 s after enhancement; *Simax*, maximum absolute enhancement; *Sirel*, maximum relative enhancement; *MRE%*, maximum relative enhancement percentage; *THR*, time of half rising; *TTP200*, time to peak within 200 s after enhancement; *Tmax*, time to maximum absolute enhancement; *NA*, not available

*Statistically significant difference between benign and malignant with *p* value < 0.05

^Statistically significant difference between borderline and malignant with *p* value < 0.05

#Statistically significant difference between benign and borderline with *p* value < 0.05

## Discussion

Pre-operative discrimination between benign and malignant ovarian masses is of major importance, particularly in children and adolescents, where preserving fertility constitutes a highly important aspect of the therapeutic approach. Although data of MR imaging from paediatric patients were scarce, this review suggests that DWI, with ADC values measured in enhancing components, and semi-quantitative DCE might increase the diagnostic performance of MR imaging in the pre-operative differentiation between benign and malignant ovarian masses.

MR imaging characteristics associated with malignancy included larger size, thicker walls, presence of septa and/or vegetation within the mass, increased signal intensity on T2-weighted imaging, increased contrast enhancement, ascites, peritoneal implants and bilaterality. This corresponds with reports in existing literature describing masses larger than 4 cm, with solid components demonstrating contrast enhancement or cystic lesions with vegetation > 1 cm (as profuse papillary projections), wall and septum thickness of > 3 mm and areas of necrosis as suspicious [52–54]. Diagnostic performance of MR imaging has a fairly good sensitivity for differentiating malignant from benign masses. Regarding specificity, however, there is still room for improvement.

DWI seems to improve sensitivity and specificity of MR imaging to 93.3–100% and 85–96.8%, respectively [25, 31]. The added value of ADC is less clear. Although ADC values for malignant masses were lower compared with benign tumours, a considerable overlap was found. This can partly be explained by ADC values depending strongly on the pathologies included, the *b*-values used and whether ADC is calculated on both solid and cystic components of the lesion, or solely solid components. Several masses of benign origin, including mature teratomas, cystic endometriosis and fibromas, might occur as false positives. These ‘complex masses’ have a more dense composition, not as a result of increased cellularity but rather as a result of the presence of keratinoid substances, products of haemoglobin degradation and dense fibres respectively [24, 25, 31]. To date, no consensus exists on which preferred *b*-value should be used in DWI of ovarian masses. When solely analysing the studies that focussed on ‘complex masses’ (excluding fat-containing lesions or solely cystic masses), using *b*-values of > 800 s/mm^2^ and calculating ADC on solid components of the mass, considerably less overlap in ADC values was demonstrated [20, 24–26, 31]. Mean ADC values for benign masses then varied between 1.16 and 1.38 × 10^−3^ mm^2^/s and for malignant masses between 0.76 and 1.03 × 10^−3^ mm^2^/s. DWI should be performed as an additional sequence in assessing non-fatty, non-haemorrhagic ovarian masses, with ADC values only measured in *enhancing* components of solid lesions, preferably with the highest *b*-value of > 800 s/mm^2^ [7]. Additionally, our results suggest an ADC cut-off of 1.1 × 10^−3^ mm^2^/s might represent the best cut-off to help discriminate between benign and malignant lesions.

Another sequence that might contribute to the specificity of MR imaging is based on the process of angiogenesis, which is characteristic of and essential to nearly all malignant tumours [11, 12]. DCE MR attempts to differentiate between benign, borderline and malignant masses by attributing them to one of the three TICs as obtained by DCE. This systematic review shows type I TICs to be fairly predictive of benign origin of the ovarian mass, whereas type III TICs are predictive of malignancy. However, the assessment of enhancement patterns remains qualitative and might therefore be subject to user bias, similar to the evaluation of masses based on morphological criteria [55]. The use of semi-quantitative parameters deducted from the TIC, for example the enhancement amplitude and time to peak, might offer a solution to this subjectivity. Unfortunately, no reliable cut-off values could be extracted due to much heterogeneity of the studies regarding the semi-quantitative parameters analysed and their corresponding cut-off values as well as diagnostic performance. TIC type alone might not be sufficient in distinguishing between benign and malignant masses, since malignant lesions such as adenocarcinomas are sometimes found to be hypovascular, whereas benign masses, e.g. thecomas or sclerosing stromal tumours, might show hypervascularity [11]. Nevertheless, the diagnostic performance of the semi-quantitative parameters seems promising and DCE-MR imaging might thus form a valuable addition. We therefore support the advice of the ESUR to consider DCE-MR imaging in inhomogeneous solid masses on T2 or in complex cystic or cystic/solid masses with concern for malignancy. To deal with the aforementioned user bias and increasing extent of the diagnostic workup of ovarian masses (by incorporating DWI and DCE as well), there might be an interesting role for radiomics to play. This ‘data-driven’ approach which enables the extraction of innumerable quantitative features from tomographic images has already shown promising results in the classification of ovarian epithelial cancer, as well as in predicting several outcome measures [56, 57]. MR spectroscopy has also been reported to play a role in differentiating between borderline and malignant epithelial ovarian tumours [58]. However, epithelial tumours are rare in the paediatric population.

This systematic review faced some limitations. Data on the performance of MR imaging, combined with DWI and DCE, were largely deducted from studies performed in adult women (with no inclusion of paediatric patients), as MR imaging descriptions by paediatric studies were insufficient and no data from a purely paediatric cohort could be obtained. However, in order to minimise the risk of missing relevant studies, such studies and case reports in paediatric patients were included. The included studies showed much heterogeneity in MR imaging protocols, which made a meta-analysis impossible.

The description of the MR imaging features of the ovarian masses was very limited in the paediatric studies, which hampers the implementation for clinical use. Previously published reviews on the imaging of ovarian masses in children and adolescents were mainly based on findings in adult women [59–62]. This systematic review attempted to select studies applicable to children and adolescents, by exclusively including studies that were conducted either on paediatric patients or on adult women where at least 20% of the included patients had a malignant ovarian tumour other than carcinoma.

In conclusion, this systematic review suggests that DWI, with ADC values measured in *enhancing* components, and semi-quantitative DCE might further increase the diagnostic performance of MR imaging in the pre-operative differentiation between benign and malignant ovarian masses. Furthermore, our data show that an ADC cut-off of 1.1 × 10^−3^ mm^2^/s might contribute to this differentiation. Prospective age-specific studies are needed to confirm the high diagnostic performance of MR imaging in combination with DWI and DCE techniques in children and adolescents with a sonographically indeterminate ovarian mass.

## Electronic supplementary material

ESM 1 (DOCX 119 kb)

## List of abbreviations

ADC: Apparent diffusion coefficient
AUC: Area under the curve
DCE: Dynamic contrast-enhanced imaging
DWI: Diffusion-weighted imaging
ESUR: European Society of Urogenital Radiology
IOTA: International Ovarian Tumor Analysis
MR: Magnetic resonance
MRE%: Maximum relative enhancement percentage
NPV: Negative predictive value
PPV: Positive predictive value
ROI: Regions of interest
SI_60_: Signal intensity at 60 s after enhancement
SImax: Maximum absolute enhancement
SIrel: Maximum relative enhancement
THR: Time of half rising
TICs: Time-intensity curves
TTP_200_: Time to peak within 200 s after enhancement
