# Supplementary material for: MR imaging in discriminating between benign and malignant paediatric ovarian masses: a systematic review
Source: Eur Radiol. 2019 Sep 16;30(2):1166–81. doi: 10.1007/s00330-019-06420-4 (PMC6957553; doi:10.1007/s00330-019-06420-4)

**Supplementary table 1 Full search strategy**

**Pubmed search**

((ovarian neoplasm[MeSH] OR ovarian neoplasm*[Title/Abstract] OR ovarian tumor*[Title/Abstract] OR ovarian mass*[Title/Abstract] OR ovarian lesion[Title/abstract] OR adnexal mass*[Title/abstract] OR ovarian germ cell tumor*[Title/Abstract] OR OGCT*[Title/Abstract] OR ovarian germ cell neoplasm[Title/Abstract] OR ovarian teratoma*[Title/abstract] OR ovarian cystic teratoma*[Title/Abstract] OR teratoma of the ovary*[Title/Abstract] OR Sex cord-stromal tumor*[Title/abstract]))

AND

(magnetic resonance imaging[Title/Abstract] OR MR imaging[Title/Abstract] OR MRI[Title/Abstract])

last performed on 26-02-2019 🡪 895 results

EMBASE search

('ovary cancer'/exp OR 'ovarian neoplasm*':ab,ti OR 'ovarian tumor*':ab,ti OR 'ovarian mass*':ab,ti OR 'ovarian lesion*':ab,ti OR ‘adnexal mass*’:ab,ti OR 'ovarian germ cell tumor*':ab,ti OR 'ogct*':ab,ti OR 'ovarian germ cell neoplasm':ab,ti OR 'ovarian teratoma*':ab,ti OR 'ovarian cystic teratoma*':ab,ti OR 'teratoma of the ovary*':ab,ti OR 'sex cord-stromal tumor*':ab,ti)

AND

('magnetic resonance imaging':ab,ti OR 'mr imaging':ab,ti OR 'mri':ab,ti) AND [2008-2018]/py

last performed on 26-02-2019 🡪 2120 results

**Supplementary Table 2. Quality assessment based on the STARD 2015 of the studies included in this systematic review regarding the use**

**of MR imaging in differential diagnosis of ovarian masses.**


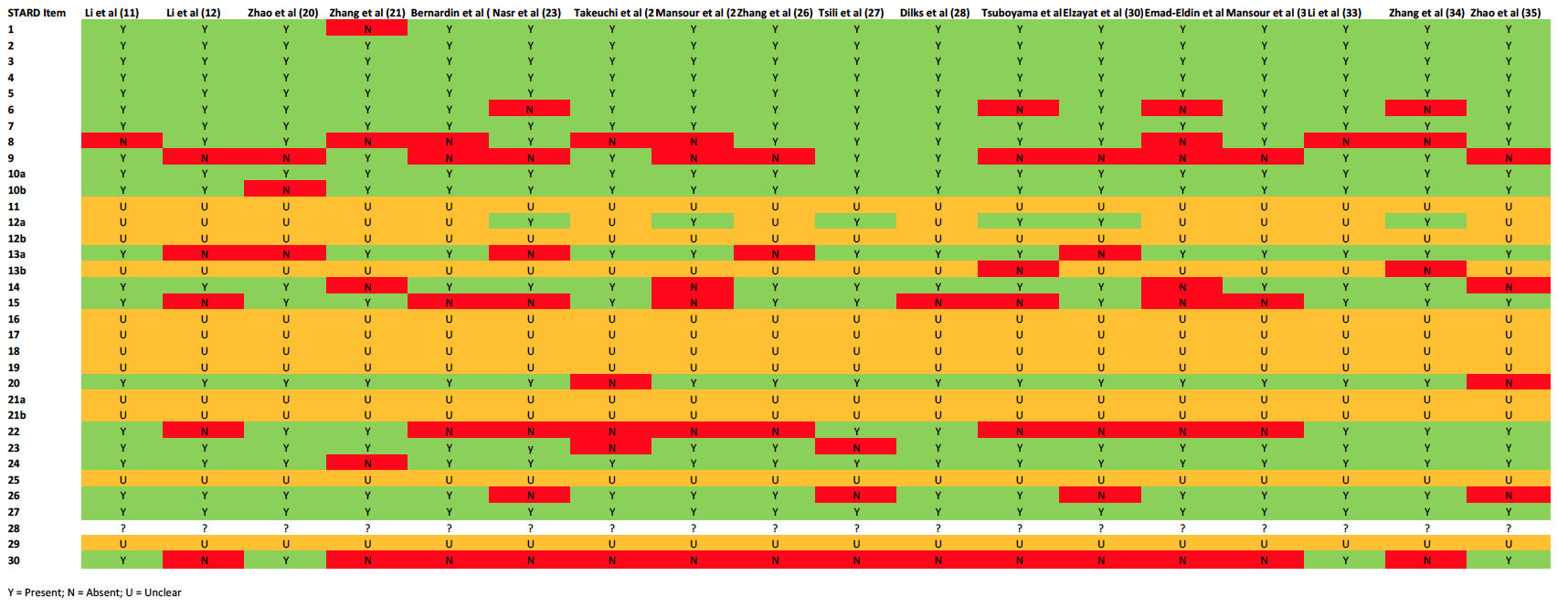

Supplement: Supplementary file 1 — (DOCX 119 kb) [file 330_2019_6420_MOESM1_ESM.docx]
